# “Anxiety, COVID, Burnout and Now Depression”: a Qualitative Study of Primary Care Clinicians’ Perceptions of Burnout

**DOI:** 10.1007/s11606-023-08536-2

**Published:** 2023-11-27

**Authors:** Debora Goetz Goldberg, Tulay Soylu, Carolyn Faith Hoffman, Rachel E. Kishton, Peter F. Cronholm

**Affiliations:** 1 Department of Health Administration and Policy, College of Public Health, George Mason University, 4400 University Drive MS IJ3, Fairfax, VA 22030 USA; 2 Center for Evidence-Based Behavioral Health, George Mason University, Fairfax County, USA; 3 Department of Health Services Administration and Policy, Temple University, Philadelphia, USA; 4 Department of Family Medicine and Community Health, University of Pennsylvania, Philadelphia, USA; 5 Department of Family Medicine and Community Health, University of Pennsylvania, Philadelphia, USA; 6 Center for Public Health, University of Pennsylvania, Philadelphia, USA; 7 Leonard Davis Institute of Health Economics, University of Pennsylvania, Philadelphia, USA

**Keywords:** primary care, professional burnout, electronic health record, qualitative research, moral injury

## Abstract

**Background:**

Clinician burnout has become a major issue in the USA, contributing to increased mental health challenges and problems with quality of care, productivity, and retention.

**Objective:**

The objective of this study was to understand primary care clinicians’ perspectives on burnout during the COVID-19 pandemic as well as their perspectives on the causes of burnout and strategies to improve clinician well-being.

**Approach:**

This qualitative research involved in-depth interviews with 27 primary care clinicians practicing in a range of settings across the USA. Semi-structured interviews lasted between 60 and 90 min and were conducted using Zoom video conferencing software between July 2021 and February 2023. Transcripts were analyzed in NVivo software using multiple cycles of coding.

**Key Results:**

Clinicians shared their experiences with burnout and mental health challenges during the COVID-19 pandemic. Contributors to burnout included high levels of documentation, inefficiencies of electronic health record (EHR) systems, high patient volume, staffing shortages, and expectations for responding to patient emails and telephone calls. The majority of participants described the need to work after clinic hours to complete documentation. Many clinicians also discussed the need for health system leaders to make sincere efforts to enhance work-life balance and create a culture of health and well-being for health professionals. Suggested strategies to address these issues included supportive leadership, accessible mental health services, and additional administrative time to complete documentation.

**Conclusions:**

The results of this study provide an in-depth view of participating primary care clinicians’ experiences and perceptions of burnout and other mental health challenges. These viewpoints can improve awareness of the issues and strategies to improve the health and well-being of our clinician workforce. Strategies include aligning payment models with the best approaches for delivering quality patient care, reducing administrative burden related to documentation, and redesigning EHR systems with a human factors approach.

## BACKGROUND

The last three years have taken a tremendous toll on the US healthcare workforce. A worldwide pandemic shut the doors of businesses, laid off workers, locked people in their homes, and separated loved ones. More than one million people in the USA lost their lives due to COVID-19^1^ and another 19 million suffer from “long COVID” symptoms.^2^ In addition to the strain on physical health, the COVID-19 pandemic increased psychological distress and is now considered to be a prominent cause of poor mental health.^3^ Health authorities, including the US Surgeon General and the World Health Organization, released statements of concern over the rise in mental health disorders, including the increase of burnout and other mental health challenges among healthcare professionals.^4–6^

At the onset of the pandemic, primary care clinicians responded quickly to the changing demand for services by transforming their model of care to provide telehealth services in addition to office-based care. During this time, clinicians faced shortages of personal protective equipment, inadequate support personnel, heightened exposure to the virus, and difficulty arranging ancillary patient care.^7^

Burnout is a syndrome of emotional exhaustion, depersonalization, and a sense of low personal accomplishment at work.^8^ Before the COVID-19 pandemic, there was a high rate of anxiety, dissatisfaction, and burnout among primary care clinicians.^9^ Recent estimates of burnout across physician specialties and experience levels indicate a dramatic increase since the start of the COVID-19 pandemic,^10, 11^ from 40 to 45% in early 2020 to 60% by the end of 2021.^12^ According to a recent report from The Commonwealth Fund, 61% of primary care physicians in the USA 54 years of age and younger and 46% of physicians aged 55 and older reported experiencing emotional distress during COVID-19.^13^ High workload, such as long working hours, complex job functions, poor communication, and administrative burdens are associated with burnout symptoms and stress.^14–16^

Moral distress, another widespread condition, is experienced when clinicians struggle to do what they believe is ethically correct. Recurrent feelings of moral distress can lead to moral injury, an overwhelming sense that an individual cannot meet their personal or professional ethical standards.^17^ While there has been increasing research on the frequency of burnout and moral injury among primary care clinicians, there is a lack of detailed information on clinicians’ experiences during the COVID-19 pandemic and their perceptions of the causes and solutions to address burnout.^18^ The objective of this qualitative study was to understand primary care clinicians’ experience of burnout during the COVID-19 pandemic, perceptions of the causes of burnout, and strategies to improve the well-being of clinicians.

## METHODS

### Study Design, Setting, and Participants

This research is part of a larger study on the adoption and implementation of health information technology and the treatment of mental health conditions in primary care practice settings. Inclusion criteria for the sample were physicians and nurse practitioners with prescribing authority who work in primary care settings. Trainees were not included in the study. There were no inclusion/exclusion criteria based on age, time in practice, or hours worked per week. We used a maximum variation sampling approach to balance clinician characteristics based on gender, age, and practice setting. Clinicians were recruited through snowball sampling and recruitment announcements posted by national associations. Recruitment of participants continued until thematic saturation was achieved and additional data did not lead to any new emergent themes.^19^ The study received approval from the George Mason University Institutional Review Board on March 2, 2021. Participants were informed of privacy and confidentiality procedures during the consent process.

### Data Collection

We conducted semi-structured, individual in-depth interviews designed to answer three research questions: (1) What are the perceptions of primary care clinicians regarding the burnout crisis during the COVID-19 pandemic? (2) What are the perceptions of clinicians on the causes of burnout? (3) What strategies are recommended by clinicians for addressing issues surrounding burnout? The research team developed an interview guide that included these questions along with follow-up probes for eliciting additional comments. Interviews were conducted by two experienced qualitative researchers (DG and TS) between July 2021 and February 2023 using Zoom video-conferencing software. Interviews lasted between 60 and 90 min. Primary care clinicians who completed the interview were compensated $250 for participation. The incentive amount was based on the expected length of the interview, originally estimated to be around 90 min.

### Data Analysis

Interview transcripts were downloaded from Zoom and cleaned by the corresponding interviewer to correct for language and grammatical errors introduced by the software. A preliminary set of codes was developed by two research team members based on the interview questions and previous literature on clinician burnout. Coding of the transcripts was then performed using NVivo by the two research team members who completed the interviews. These data analysts met several times to discuss the coding process, data interpretation, evolution of the coding scheme, and the emergence of key themes. We used an integrated approach that involved several cycles of coding and analysis.^20^ We developed and applied deductively derived codes based upon our conceptual model as well as inductively derived codes that emerged from a close reading of the interview transcripts.^21^ A physician qualitative researcher on our team performed a secondary review of key themes and corresponding quotes to evaluate whether there was sufficient evidence to substantiate themes. Disagreements were resolved through consensus.

## RESULTS

Study participants included 27 primary care physicians and nurse practitioners from across the USA. Participants worked in a range of practice settings and rural-urban-suburban locations. Participant characteristics are listed in Table 1.

1. Clinician Experiences with Burnout

**Table 1:** Participant Characteristics (*N*=27)

*Characteristics of participants*	*N (%)*
Clinician type	
Physician	24 (89%)
Nurse practitioner	3 (11%)
Participant age group (years)	
25 to 34	6 (22%)
35 to 44	7 (26%)
45 to 54	4 (15%)
55 to 64	8 (30%)
65 and older	2 (7%)
Gender	
Female	16 (59%)
Male	11 (41%)
Practice setting	
Academic medical center	13 (48%)
Private practice, academic affiliation	6 (22%)
Private practice, no academic affiliation	5 (19%)
Health system practice	2 (7%)
Community health center	1 (4%)
Location	
Urban	17 (62%)
Suburban	5 (19%)
Rural	5 (19%)
United States Region	
South	16 (59%)
Northeast	8 (30%)
West	2 (7%)
Midwest	1 (4%)

Clinicians shared their experiences over the last few years, with key themes centering on increased mental health challenges involving professional burnout, exposure to trauma, and moral injury. Key themes and corresponding quotes are provided in Table 2.

**Table 2:** Themes and Examples of Supporting Quotes

Theme	Supporting quotes	Participant ID
1. Provider experience with burnout		
Accelerated burnout and mental health challenges	“…with the pain and like mourning any loss. I went back into counseling. I lost, in the space of 3 weeks, I lost 4 of my patients.”	MD65+M#7
	“…I’m reflecting for myself about this pattern of waxing and waning levels of burnout… I hear that for others, too. And, yeah, COVID, has just made it all worse.”	MD45-54M#10
Increased workload	“We have added in a patient slot for each session. So, a morning session and afternoon session each day… that was not there previously.”	NP35-44F#4
	“As I’ve stayed with the practice, the appointment times have gone down to, you know, 15 minutes, so less time. A couple of providers have left the practice.”	MD25-34F#2
Increased staffing issues	“They realize that this is an issue, but because of COVID and the lack of personnel… I don't know what they're able to do.”	MD55-64F#25
	“COVID has just made it all worse. And staffing is huge… The really good ones [medical assistants] are moving on to other things, and we're not getting new ones. And so, it's really, it's really tough.”	MD45-54M#10
2. Contributors of burnout and stress		
Inefficient electronic health records	“The electronic health record is the largest source of burnout because people end up spending a lot of time in there… that's both in the office and outside the office, at home.”	MD55-64M#15
	“The bottom-line problems. You know what I’m talking about. The user interface is terrible, and it doesn't have multiple windows at the same time, it doesn't have an efficient workflow.”	MD65+M#17
Increasing levels of patient emails and telephone calls	“You kind of have to clean out that inbox, and you don't get to it. And that creates a layer on layer of stress in your mind, you know, like, okay, I’m not going to get to messages.”	MD55-64F#25
	“I get a message about a paragraph long from a patient who has heartburn for two days and asked me what to do… you have to deal with these like constant bombardment, and that is my burnout.”	MD25-34F#11
High level of documentation	“There is this phrase ‘death by a thousand clicks.’ So, you know, the EMR really was meant to supposedly help people... but now it's being used more for just billing purposes, and so much in the in basket is causing stress and burnout.”	MD55-64F#23
	“I have not found a clinician yet that doesn't complain about their EHR and technology demands. So, I think fundamentally that [high level of] documentation, EHR, and all this is a major driver of burnout.”	MD45-54M#1
Work after clinic	“I have a colleague who comes in at seven, eight in the morning, and then doesn't leave. He's an extreme case because he doesn't want to do any work at home. I don't like to stay in the office that much, let me have the dinners with family. Yeah, but after dinner, then I’m doing the work.”	MD55-64F#14
	“Work life separation essentially, you're always like able to see stuff. You know, like on Saturday, I find that I’m always checking labs, I checked Saturday morning, and again on Saturday night.”	MD35-44F#3
Time constraints	“When seeing our adults, we just need that time. And so, the thought of adding on more patient slots with that kind of really medically complex [patients].”	NP35-44F#4
	“Primary care is already extraordinarily stressed and time crunched.“	MD35-44M#19
3. Recommended solutions to address burnout and stress		
Team-based care	“I think team-based care is very helpful. Knowing that this is your group of patients and this is the team that's going to be managing them. And the patients realize this is my team.”	MD55-64F#25
	“Get on board with teams and empowering other people to do it. And, so, I think it's huge. But again, the key is that you've got to keep a team together.”	MD45-54M#10
Institutional recognition	“Foundational is institutional recognition that [burnout] is going to be present at some level for every clinician... just let clinicians know that there's a space and room for people to feel that and then, of course, to come to someone to talk about it.”	MD35-44F#3
	“Acknowledging is so big right because so much of this, this pandemic has brought it to light right now. The system is not made for the providers at all.”	MD25-34F#11
Optimizing EHR functionality	“Thoughtful design… and meaningful user acceptability testing. So, testing every part and making it as useful as possible, time saving and beneficial.”	MD55-64M#15
	“If the ease-of-use factor is high, and then I think that can help with the moral injury and burnout.”	MD35-44F#3
Reducing documentation	“Paperwork, I mean from a primary physician standpoint paperwork is the bane of our existence… insurance and FMLA is the thing that I fill out all day, every day.”	MD35-44M#26
	“We need to have unified electronic medical records, unified reporting systems. We need to get rid of the administrative burden which is a MacArthur of problems.”	MD65+M#17

Information in participant ID column indicates provider type, age, gender, and participant number

“Anxiety, COVID, burnout and now depression,” was how one physician described her experience. “It’s not a doctor’s natural inclination… [to say to yourself] ‘you’re burned out.’ I think it’s really the events over the last three years, since COVID that tipped me over the edge.” She then opened up about her mental health struggles, “I personally developed clinical depression about a year and a half ago, the first time in my life, and it sort of spiked again this summer, and that is the result of burnout” (MD55-64F#18). Another physician, who was in her second year of residency during the pandemic, explained, “I was right on the front lines doing ICU care and holding people’s hands at the end of life. It was horrible!” (MD25-34F#11). Other clinicians described pre-COVID-19 experiences with mental health challenges in which they had to “revisit” a counselor during the pandemic, because as one physician described, “the pain has just been so bad” (MD65+M#7), as a result of losing patients to COVID-19.

Other participants described experiencing moral distress or moral injury resulting from organizational policies or operating procedures that do not align with quality patient care or work-life balance. One physician from a healthcare system stated, “I have a ton of patients who I know exactly what they need, but I don’t have the resources or time to provide it” (MD35-44M#19). Others attributed emotional harm to system-level factors such as a disconnect between fee-for-service reimbursement models and quality patient care. One physician reflected on the system as “an industry that is treated like you’re buying clothes or groceries when you have to treat the patient or else they will die” (MD25-34F#11).

Despite the high number of clinicians in our study who described their struggle with burnout and other mental health challenges, most participants also displayed a sense of hopefulness and optimism. One physician stated, “I recognize now that I’m burned out and I’m trying to take steps because I still see myself practicing medicine for the next ten years, and I want to be happy doing that” (MD55-64F#18).

2. Contributors of Burnout and Stress

Clinicians in our study shared their perspectives on the largest contributors to burnout, which included inefficiencies with electronic health record (EHR) systems, high levels of documentation, increased expectations for communicating with patients outside of visits, and increased workload due to staffing shortages and productivity requirements. These issues often required clinicians to work after clinic hours.

Many clinicians attributed the EHR as one of the main causes of burnout in primary care. One clinician explained, “It's been a very painful process over the last twenty years, and I absolutely think the electronic medical record leads to burnout” (MD55-64F#18). This viewpoint was shared by the majority of clinicians aged 55 years and older, while younger clinicians often described the EHRs as a “double edged sword” that has both good and bad characteristics. Most clinicians under the age of 55 described positive traits of EHRs and other digital technologies that help these clinicians treat patients. All participants, however, described the inefficiencies of their EHR systems and made comments such as “death by a thousand clicks” (MD55-64F#23) and “numerous hard stops” (MD55-64M#9). Most clinicians, regardless of age or provider type, described the need to work after clinic hours to complete medical record documentation. Many clinicians also shared their frustrations over the increasing volume of patient emails, texts, and telephone calls they receive.

Other commonly reported difficulties during the COVID-19 pandemic were increased workload and increased staffing issues as a result of shortages of providers, nurses, and administrative staff. Numerous participants described an increased demand from their healthcare delivery system for primary care clinicians to see more patients during the pandemic. As one physician stated, “[They’re] trying to fix some of the financial shortfall from the health system by telling clinicians to see more patients… And then they’re surprised. People burnout or leave” (MD45-54M#20).

There were also a number of issues discussed by clinicians in our study that were not widespread, yet deserve mention. Several clinicians described feeling undervalued by their health system because of the lack of inclusion in decision making, high workload, or low compensation.

3. Recommended Strategies to Address Burnout and Stress

Clinicians in our study described a range of strategies with how to address burnout and mental health challenges experienced themselves or by other health professionals in their practice. The most commonly discussed strategies were at the organizational level and included enhancing the use of team-based care models, optimizing EHR functions, and increasing institutional awareness and recognition of the issues surrounding burnout. Many participants also identified the need for additional administrative time to complete medical record documentation.

Preventive strategies were discussed by one clinician in an independent practice. This physician described her efforts to create a culture of well-being and prevent burnout among clinicians, stating, “Wellness and anti-burnout measures have been a priority of my practice since the day we were founded. We’ve made a lot of decisions, which you know cost money and resources, but we feel it’s really important that we direct our funds in this way to prevent burnout of our providers” (MD55-64F#18). These strategies included hiring a national call system that uses telephone triage to address 90% of patient needs after hours. Practice scheduling policies allow 60 min for annual wellness visits and 30 min for regular exams to ensure clinicians have enough time to address patient concerns and provide quality patient care. The practice also has dedicated NPs that cover other clinicians’ patients during their days off. As a result of recent provider feedback, the practice now allows clinicians to work from home one day a week to conduct telehealth patient visits.

Many clinicians discussed the need for health system leaders to recognize issues with burnout and to make sincere efforts to enhance work-life balance and create a culture of health and well-being. This includes supportive leadership and accessible mental health services along with time away from work to access these services. One young physician noted that what helped her during the pandemic was to have colleagues check in and ask, “How are you feeling about this? If you need to take a mental health day or something… it is okay” (MD25-34F#21). Along with the notion of work-life balance, several clinicians mentioned a need for time off with someone covering their patients.

At a systems level, most clinicians discussed the need for reducing administrative burden associated with billing requirements and quality reporting. Several physician participants also described the long-standing professional culture in medicine that supports long-working hours and personal sacrifice, which they suggested needs to change to center on clinician health and well-being. One physician stated “I don’t think that American culture is very good at promoting work-life balance. Then, on top of that, the medical culture glorifies martyrdom” (MD45-54M#10).

Few participants in our study mentioned strategies at the individual level to address burnout. Several clinicians, however, indicated a high level of professional satisfaction in their role as a primary care provider and a deep connection between their work and the mission of the organization as conditions that help them overcome stressful situations. As one provider explained, “I really love our clinic… We’re all very unified in our mission, and who we’re serving, and that helps a ton” (NP35-44F#4). While no clinicians indicated an intention to leave their current practice, there were three female clinicians who explained their previous struggles with burnout and the difficult decision they made to leave their practice and start new positions that offered more autonomy, flexible working arrangements, and opportunities to provide better patient care.

## DISCUSSION

Clinicians participating in our study shared their experiences with burnout and other mental health challenges during the COVID-19 pandemic. Numerous clinicians reported experiencing traumatic events during the pandemic and in some cases reported both burnout and depression. Our study highlights the organizational and system contributors to burnout.

Primary care delivery exists in a rapidly changing environment characterized by continuously evolving technology, regulatory policies, and care delivery models that have accelerated the incidence of burnout and depression among primary care clinicians.^22^ One of the principal findings from our study was the overwhelming belief by clinicians that inefficiencies of EHR systems and high levels of documentation contribute to burnout. Thirty years ago, EHR systems were envisioned as a tool to meet the needs of clinicians for patient care. At that point in time, it was thought that clinician users would be involved in system design and key features of EHRs would include local control and customization,^23^ fundamental strategies that failed to be implemented. EHR systems were designed by engineers and are driven by billing requirements, not necessarily the needs of clinicians or patients. EHR vendors produce out-of-the-box, standardized systems that prevent clinicians from efficiently completing medical record documentation, often requiring clinicians to create work arounds or spend considerable time completing medical records. Recent changes from the Centers for Medicare and Medicaid Services (CMS) may help mitigate ambulatory care documentation requirements.^24^ To support clinicians, EHR vendors and the healthcare systems that purchase these systems need to pay special attention to human factors related to clinical workflow, such as building flexibility into the system so that clinicians can configure the EHR to fit the workflow in their practice.

Other contributors to burnout included a high patient volume and an increase in direct patient communication through texts and emails. Most clinicians described a need to work after clinic hours to return patient messages and complete medical record documentation. Our findings align with previous research that reported primary care physicians indicated dramatic increases in their workload since the beginning of the pandemic ^25, 26^ and an escalation in staffing issues, both contributing to an increase in burnout and stress among clinicians. A few participants in our study felt they were undervalued and received a lack of support from health system leaders, similar to previous studies.^27^

At the organizational level, efforts should be taken by health systems and practice leaders to create a working environment characterized by engaged leaders, strong communication, and a team-based care model. A supportive work environment centered on the health and well-being of clinicians and other health professionals is a necessary foundation for delivering quality patient care. Previous studies support the strategies suggested by our study participants to enhance provider well-being. One study conducted in a large health system resulted in recommendations to increase staffing, increase administrative time, improve practice and leadership communication, and enhance teamwork in primary care practices.^28^ Clinicians who work in team-based care models and work environments characterized by supportive leadership and strong communication experience less anxiety and other mental health challenges.^29, 30^

Systems-level contributors to burnout are the hardest to address. The government can support efforts to optimize use of health information technology by funding research and development of digital technologies that engage clinicians and patients in the design or redesign of these systems.^31^ Payers can also play an important role in reducing provider burnout by decreasing administrative burden related to documentation and reporting requirements. Equally important is payment reform. Payers need to adequately reimburse primary care clinicians to support their role in caring for complex patients such as individuals with multiple chronic health conditions, behavioral health needs, or social and economic hardships. Increased reimbursement would allow practices to hire additional staff to provide patient education, coordination of care, and assist patients with other needs.^32, 33^

### Limitations

We experienced challenges in recruiting primary care clinicians for this study despite the $250 incentive to participate. Clinicians who responded to our recruitment announcement but declined to participate stated “lack of time” as the reason for not participating. We recruited a high number of clinicians from the northeast and southern states and a high number of clinicians in academic settings, which may have introduced a biased discussion of challenges experienced in those settings that do not exist in other primary care settings. Our qualitative approach and sampling issues limit interpretation of the findings to hypothesis generation. We did, however, include methods to improve the credibility, confirmability, dependability, and transferability of the findings, which include the use of a semi-structured interview guide, a multidisciplinary team for data analysis, secondary review of themes by a physician qualitative researcher, and maintenance of an audit trail of theme development and analytic decisions.^34^ The results of this study provide an in-depth view of participating clinicians’ experiences and perceptions of burnout and other mental health challenges. These viewpoints can improve awareness of the issues and strategies to improve the health and well-being of our clinician workforce.

## CONCLUSION

Our findings highlight the importance of a systems approach to support the health and well-being of clinicians with interventions at the individual, organizational, and systems levels. Organizations can acknowledge the issues surrounding clinician burnout and build supportive working environments characterized by engaged leaders, strong communication, and a team-based care model. Investments at the organizational level to build a supportive work environment and an organizational culture of health, safety, and well-being could reduce costs associated with turnover and lost revenue linked to decreased productivity.^35^ Key strategies to reduce burnout include aligning payment models with the best approaches for delivering quality patient care, reducing administrative burden related to documentation and reporting requirements, and redesigning EHR systems with a human factors approach.
